# Why the spaces in which we deliver care matter: implications and recommendations for general practice

**DOI:** 10.3399/bjgp24X738741

**Published:** 2024-06-28

**Authors:** Lindsey Kent, Rebecca Goulding, Jennifer Voorhees, Jonathan Hammond, Jessica Drinkwater

**Affiliations:** Faculty of Biology, Medicine and Health, School of Health Sciences, University of Manchester, Manchester.; Faculty of Biology, Medicine and Health, School of Health Sciences, University of Manchester, Manchester.; Faculty of Biology, Medicine and Health, School of Health Sciences, University of Manchester, Manchester.; Faculty of Biology, Medicine and Health, School of Health Sciences, University of Manchester, Manchester.; Faculty of Biology, Medicine and Health, School of Health Sciences, University of Manchester Manchester; visiting research fellow, Leeds Institute of Health Sciences, University of Leeds, Leeds.

## Introduction

The physical spaces in which primary healthcare is delivered matter and have the potential to profoundly affect both patients and staff. This potential is illustrated by academics, who describe the connection between the spaces in which care is delivered and how cared for, and well, patients feel.^1^^,^^2^^,^^3^ Considering healthcare space as a *‘passive background’*^1^ underestimates its impact.^1^ Instead, conceptualising space as ‘active’ better describes its role as ‘participative in the very making of care and health’.^1^ While many existing studies focus on hospital settings, there is evidence to suggest that the same is true for primary care.

During the COVID-19 pandemic, there were dramatic changes to general practice spaces, and the negotiation of such spaces, in the UK. These changes (including locked doors, spatial protocols for queueing or sitting, and remote consulting), which continued to evolve as the pandemic did, had a variety of consequences for patients and staff. Many patients felt shut out of their surgeries, but patients from diverse backgrounds and those with long-term or complex health needs were disproportionately exposed to the negative effects of such changes.^4^ At the same time, NHS England and the Royal College of General Practitioners (RCGP) recognised that there is a shortage of space to deliver a needed increase in capacity to meet rising patient demand, house additional staff, and support teaching and training opportunities.^5^^,^^6^ Ensuring suitable spaces exist for collaborative care delivery is a necessity for meeting various national policy goals as well as ‘opening the door’ for improved connections between patients and their GP surgeries. This article considers the ways in which focusing on primary care spaces could lead to improved outcomes from the perspectives of both patients and staff.

## Understanding the variety and complexity of general practice spaces

GP surgeries have evolved over time, each from a unique starting point. This is reflected in the diversity of building stock in use, ranging from modified family homes to multi-provider clinics, all covered by a variety of contract arrangements.

Current funding for GP premises improvement comes from surgeries self-funding or applying for funds from the NHS. There is a significant shortfall, which the RCGP estimates at £2 billion, needed to address GP infrastructure issues, with two out of five general practice staff saying their premises are not fit for purpose.^6^ NHS England has made addressing this a priority area,^5^ but also admits that they do not have a clear understanding of the current general practice estate, and this is a significant barrier to long-term planning.^5^ Primary Care Networks (PCNs) have been encouraged to undertake a mapping exercise to develop neighbourhood-level estates strategies, linking population need to future estate development.^7^ Additionally, a long-awaited update to the Premises Costs Directions (PCD) has just been released.^8^ This details the payment arrangements and funding that general practices can expect for premises improvements. However, there is limited detail on where this funding will come from, with no new money to cover the identified shortfall. Thus, it may only happen if funds can be found from existing estate budgets in other areas of the NHS. The impact of the updated PCD will take time to be felt, and further guidance will be released in the coming months.

To make the argument for further funding, it is vital to understand why general practice spaces are so important and diverse. Surgeries are complex spaces that include communal areas (waiting rooms, corridors, and bathrooms) and behind-the-scenes areas (offices and storage rooms), in addition to consulting rooms. As well as the physical contents and organisation of a room or area (for example, furniture, lighting, wall coverings, materials, and layout), spaces also comprise the people within them and the sounds, smells, and other stimuli present. When defining space, it is also important to consider how being in a space can make people feel. Van der Meide explores this idea of *‘perceptual experience’* through the concept of *‘lived space’*:

*‘… lived space is difficult to put into words and yet we know that the space in which we find ourselves affects the way we feel. The huge space of, for example, a train station may make us feel exposed and small, and a nice and cosy restaurant lets us feel at ease. The typical (sterile) air we smell when we enter the hospital can reassure us or instil fear.’*^9^

## How could addressing space improve the care we provide?

Ulrich’s supportive design theory builds on the subjective and perceptual nature of inhabiting a space described by van der Meide. It is based on the idea that a healthcare environment could reduce stress if it engenders a perception of control, social support, and positive distraction. These three elements promote wellness and aid recovery by shaping healthcare spaces to be *‘psychologically supportive’* and *‘complementary to the healing effects of drugs and other medical technology’*.^3^ This is relevant to GP surgeries, where patients may feel stressed because of undifferentiated symptoms, and the fear of potential diagnoses. They may anticipate having a difficult interaction with a receptionist or be facing a painful test or exposing clinical examination. Their journey to accessing an appointment may have left them feeling powerless, frustrated, or anxious, and this stress may be shared by the carers or relatives who attend with them. Staff are also affected by stressful surgery environments, as they strive to provide good care in a system that is stretched and under-resourced, and over which they may lack control. Applying supportive design principles may address these issues and reduce psychological distress for both staff and patients by creating an environment in which good care is both easy to provide and receive.

Evidence for supportive design and its clinical impact is growing. Ulrich described supportive design’s potential to reduce hypertension, anxiety, and pain, and also noted that poor design could increase delirium, pain, and low mood.^3^ Evidence from hospital settings demonstrates that aspects such as furniture arrangements and noise can effect clinical outcomes and physiological parameters. Devlin and Arneill found that waiting room features, such as warm lighting, quality furnishings, and the presence of artwork, led to a higher perceived quality of care.^2^ Improving the design and atmosphere of a sexual health clinic reinforced expectations of privacy and care, and alleviated worries or concerns.^10^ Bernhardt *et al*’s 2022 review of hospital design for stroke care noted this too when describing how *‘corridor design, and staff station placements can all impact care provision, staff and patient behaviour’*.^11^ These findings about supportive design theory are likely to be applicable across all healthcare settings. One study exploring changes during COVID-19 found that, in general practices too small to support infection control measures, patients were kept outside and communicated with via intercom. This created additional clinical risk and less caring interactions that could negatively affect both patients and staff.^12^ Staff also lost access to communal spaces to rest and had fewer informal interactions with other staff and patients, impacting their wellbeing and ability to deliver quality care. In addition, there is evidence that interior architecture in primary care can affect interpersonal interactions and collaboration between staff.^13^ In designing healthcare premises, organisations have an opportunity to recognise the importance of providing spaces that support staff to deliver high-quality care, and co-designing spaces (with staff and patient users) could reduce stress and increases efficiency.^14^^,^^15^

Despite this, examining the design of healthcare spaces is a neglected area of research and policy in UK general practice.^16^ Ulrich emphasises that, if evidence is lacking, decision makers and designers should employ *‘solutions that promote wellness’* and improve healthcare outcomes.^3^

## Considerations for policymakers and researchers

Ulrich’s supportive design theory could benefit healthcare outcomes, staff retention, and job satisfaction, which may reduce healthcare costs, increasing the argument for investment. Applying its principles is likely to facilitate provision of high-quality care. This may prove important when developing a primary care estates plan, especially in the context of pressured budgets, and would be a motivator for GPs and others working in primary care spaces. It may also be a potential policy lever for NHS decision makers given pandemic-related pressures and the likelihood of ongoing funding challenges.^8^ It is essential that a review of premises funding take place, especially in the context of the new PCD. National implementation of this should include an inequalities impact assessment to ensure equity in funding distribution, and local implementation should be co-designed with both patients and staff. Concurrently, transdisciplinary research focused on UK general practice spaces is urgently required to investigate the best design processes and the impact of space changes on patients and staff.

## Recommended approaches for general practice

General practices and PCNs currently designing or upgrading their spaces and estates strategies should ensure regular feedback is sought from patient and staff groups or their representatives.^12^ To ensure that spaces promote healing over hostility, the needs of patients and staff must be the primary motivating factor behind any new strategy. There is also a need to work with experts in healthcare design and space management, rather than generic consultancy firms, whose remit may not include aspects of this specialism.^15^

The following are practical recommendations, based on a review of limited existing data relating to general practice, and the likely applicability of findings in the hospital literature to other healthcare contexts.^16^ These recommendations stem from the notion that space design can transmit a message of care and the values of the general practice team, as well as improve wellness and healthcare outcomes:

- *Minimise or avoid physical barriers*. This can reduce aggression and facilitate communication between staff and patients, promoting more caring and therapeutic interactions;^17^
- *Create calming public and private spaces*. This could include private individual seating, ideally with nature views or art. Some patients may wish to be in spaces with good ventilation, or visible air filtration, because of concerns about virus transmission;^18^
- *Accept duality*. Some patients and staff will favour a clinical, efficient-feeling space, and others a warm and homely one. Consider which may appeal most or include both elements where feasible;^2^
- *Interactive areas*. Spaces in which patients can engage, interact, or control an aspect of the space may help reduce stress and promote positive social interaction, which can help mood and pain management.^3^ This could include a blood pressure or health check machine or a way of adjusting lighting or volume in an area. Some patients will enjoy a space that facilitates opportunistic interactions with others;
- *Images and messaging that empower or offer information in an accessible way*. Consider the language used in health promotion messages and the impact of negative images (such as depictions of injury/disease) in a place of care;^19^
- *Clear signage, good lighting, and reduced clutter*. This should help patients orientate themselves to the space and its processes, improve their perceptions of the service they receive, and reduce anxiety; and^20^
- *Break space*. Communal spaces for staff should allow moments of respite from tasks, and spaces that encourage informal interaction can encourage collaboration and job satisfaction.^13^

## Conclusion

Estates issues are high on the agenda of NHS England and PCNs, making it an ideal time to consider the wider impacts of healthcare spaces on key outcomes. It is vital that the design of primary care estates moves from a ‘making do’ approach to one that is driven by supportive design theory with the potential to improve the wellbeing and healthcare needs of patients, and the ability of staff to deliver excellent care. With a new PCD in place, an opportunity exists to revolutionise surgery spaces and benefit those who use them. We ask that all primary care staff seek out their estates strategy lead (via their practice manager or within their PCN) and share their perspective on how changing healthcare spaces could bring benefits and rewards. There may be lessons to learn from the GPs who worked from their homes in the past — if we invest in our healthcare spaces with as much care as we do the design of our own homes, we could create spaces that better meet the present and future needs of primary care.
